# Cathelicidin, an antimicrobial peptide produced by macrophages, promotes colon cancer by activating the Wnt/β-catenin pathway

**DOI:** 10.18632/oncotarget.2845

**Published:** 2014-12-15

**Authors:** Dong Li, Wenfang Liu, Xuan Wang, Junlu Wu, Wenqiang Quan, Yiwen Yao, Robert Bals, Shurong Ji, Kaiyin Wu, Jia Guo, Haiying Wan

**Affiliations:** ^1^ Department of Clinical Laboratory, Tongji Hospital of Tongji University, 200065 Shanghai, China; ^2^ Department of General Surgery, Tongji Hospital of Tongji University, 200065 Shanghai, China; ^3^ Department of Pharmacy, Putuo People's Hospital, 200060 Shanghai, China; ^4^ Department of Internal Medicine V – Pulmonology, Allergology, Respiratory Intensive Care Medicine, Saarland University Hospital, 66424 Homburg, Germany; ^5^ Institute of Pathology, Charité University Hospital, 12200 Berlin, Germany; ^6^ Tongji University Suzhou Institute, 215000 Suzhou, China

**Keywords:** Cathelicidin, Colon cancer, Macrophage, Wnt/β-catenin, Growth

## Abstract

Here we found that levels of cathelicidin, an antimicrobial peptide, were increased in colon cancer tissues compared to noncancerous tissues. Importantly, cathelicidin was mainly expressed in immune cells. Contact with tumor cells caused macrophages to secrete cathelicidin. Neutralization of cathelicidin, *in vivo*, significantly reduced the engraftment of macrophages into colon tumors, as well as proliferation of tumor cells, resulting in an inhibition of tumor growth. Furthermore, treatment with cathelicidin neutralizing antibody de-activated the Wnt/β-catenin signaling pathway in tumor cells both *in vivo* and *in vitro*. Cathelicidin activated Wnt/β-catenin signaling by inducing phosphorylation of PTEN, leading to activation of PI3K/Akt signaling and subsequent phosphorylation of GSK3β, resulting in stabilization and nuclear translocation of β-catenin. These data indicate that cathelicidin, expressed by immune cells in the tumor microenvironment, promotes colon cancer growth through activation of the PTEN/PI3K/Akt and Wnt/β-catenin signaling pathways.

## INTRODUCTION

Clinical evidence indicates that chronic inflammation is linked with cancer development [1]. For instance, ulcerative colitis (UC) is an inflammatory bowel disease that is associated with colorectal cancer (CRC) [2]. Emerging evidence indicates that numerous soluble factors secreted by infiltrating immune cells contribute to cancer growth, including the growth and progression of CRC [1, 3–5]; however, the actual role of these molecules in tumor promotion is poorly understood.

The human cathelicidin gene, *CAMP*, is isolated from human cells and codes for human cationic antimicrobial peptide-18 (hCAP-18) [6]. HCAP18 consists of 3 domains: a signal peptide, a cathelin domain and an LL-37 peptide [7]. The LL-37 peptide is maintained in its pro-peptide form until secretion by cleavage of protease3 [7, 8]. *CAMP* is constitutively expressed by specialized host defense cells, including macrophages, neutrophils, epithelial cells of the skin, and endothelial cells on the gastrointestinal, urinary and respiratory tracts [6, 9]. The *Camp* gene codes for the cathelicidin-related AMP (CRAMP) peptide in mice, which is similar to the LL-37 peptide in humans. Both have α-helical structures, antimicrobial functions and analogous tissue distribution [6, 7]. In addition to combating microorganisms, cathelicidin plays a role in various immune functions, including immune modulation, inflammatory reactions, cell proliferation, angiogenesis and inhibition of apoptosis [8, 10].

Emerging evidence suggests that cathelicidin is involved in the promotion of tumor growth [11, 12]. Up-regulation of LL-37 has been observed in human lung cancer and administration of synthetic and biologically active LL-37 peptide, or transgenic expression of LL-37 in tumor cells increases lung tumor cell proliferation [9]. Knockdown of *Camp* in myeloid cells decrease the tumor proliferation and inflammatory cell recruitment of in a murine lung cancer model [6]. In ovarian cancer, LL-37 contributes to cell proliferation, invasion and cancer progression through direct stimulation of tumor cells, initiation of angiogenesis and recruitment of immune cells [7, 13, 14]. Surprisingly, it has been reported that the expression of LL-37 was downregulated and treatment with LL-37 caused cell-cycle arrest and tumor cell apoptosis in gastric adenocarcinomas [15].

In this study, we collected human colon cancer tissues and established colon cancer mouse models. We aimed to examine the expression of cathelicidin and its pathogenic effects in the colon cancer, and identify the underlying molecular mechanisms.

## RESULTS

### Expression of cathelicidin in human colon cancer tissue

**Figure 1:**
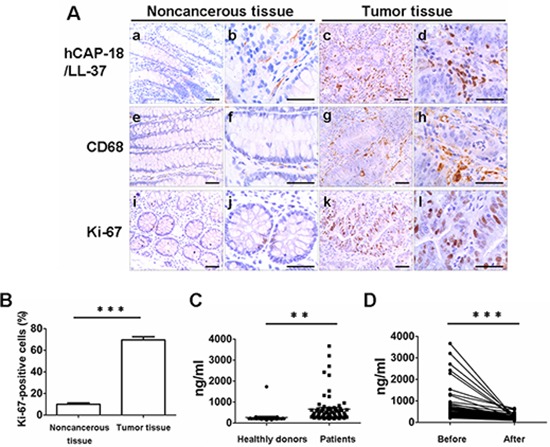
Human colon cancers express cathelicidin, exhibit accumulation of macrophages and show strong tumor proliferation **(A)** Immunohistochemical analysis of LL-37 expression in noncancerous colon tissue (a, b) and colon cancer tissue (c, d). Representative macrophage marker CD68 (e–h) and Ki-67 (i–l) in (e, f, i, j) noncancerous tissue and (g, h, k, l) cancerous tissue. Scale bar in a, c, e, g, i and k = 100 μm; in b, d, f, h, j and l = 50 μm. **(B)** Percentage of Ki-67-positive tumor cells. Results are mean α SEM, ***p < 0.001. **(C)** Concentration of hCAP-18/LL-37 in the serum of colon cancer patients and healthy humans was measured by enzyme-linked immunosorbent (ELISA) assay. **p < 0.01. **(D)** Concentration of hCAP-18/LL-37 in the serum of patients with colon cancer before and after surgery was measured by ELISA assay. ***p < 0.001.

Human colon tumor samples were analyzed by immunohistochemistry. Sections of noncancerous colon tissue showed weak staining for hCAP-18/LL-37; however, colon cancer tissue sections showed strongly positive staining for hCAP-18/LL-37. A total of 60/68 (88.2%) colon cancer tissue samples were positively stained (Fig. 1A, a–d). Interestingly, the expression of hCAP-18/LL-37 in tumor cells and colonic epithelial cells was considerably weak and almost un-measurable, whereas infiltrating inflammatory immune cells in the stroma expressed remarkably higher levels of hCAP-18/LL-37 (Fig. 1A, a–d). Macrophage infiltration in tumor tissue from patients was examined via CD68 immunostaining. There were few macrophages that were positive for CD68 in noncancerous colonic mucosa; however, there was a large number of CD68-positive macrophages in tumor regions (Fig. 1A, e–h). In addition, the number of cells that were positive for the proliferation marker, Ki-67 was higher in tumor tissue than in noncancerous colon tissue (Fig. 1A, i–l, 1B). These data indicate that hCAP-18/LL-37 is highly expressed in human colon cancer and that infiltrating inflammatory immune cells are the main source of hCAP-18/LL-37 in tumor tissue.

Serum levels of hCAP-18/LL-37 were also measured in patients diagnosed with colon cancer. Consistent with the changes in expression of hCAP-18/LL-37 in tumor regions, the concentration of hCAP-18/LL-37 in the serum was much higher in patients with colon cancer than in healthy humans (Fig. 1C). Blood levels of hCAP-18/LL-37 were evaluated in patients with colon cancer, both before and after surgery, to determine whether the higher level of hCAP-18/LL-37 seen in patients with colon cancer was due to the presence of tumors. Patients had significantly decreased levels of cathelicidin in the blood 1 month after surgery compared to before surgery (Fig. 1D). These results are consistent with those obtained by immunohistochemical analysis of human colon tumor tissue.

### Macrophage-derived cathelicidin accelerates the growth of colon cancer cells *in vitro*

**Figure 2:**
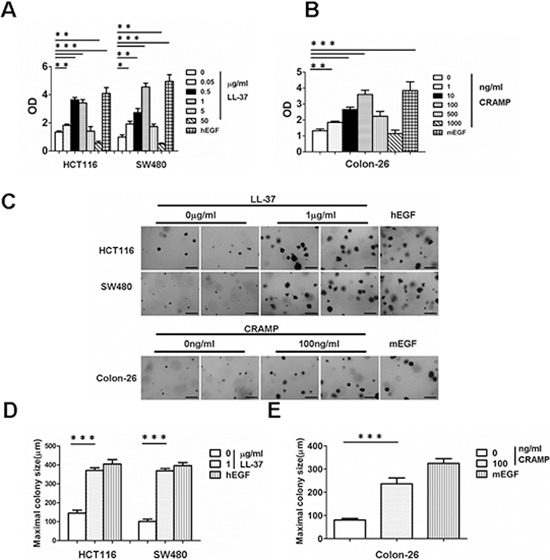
Exogenous cathelicidin increases proliferation and soft agar colony-formation in colon cancer cell lines HCT116, SW480 and Colon-26 **(A)** Human colon cancer cell lines, HCT116 and SW480, were treated with the indicated doses of LL-37 or 100 ng/ml human EGF for 96 h at which time cell proliferation was measured by ELISA (BrdU labeling) analysis. **(B)** Mouse colon cancer cell line, Colon-26, was treated with the indicated doses of CRAMP or 10 ng/ml mouse EGF for 96 h and cell proliferation was measured by ELISA (BrdU labeling) analysis. Results are mean α SEM, *p < 0.05; **p < 0.01; ***p < 0.001. **(C)** HCT116, SW480 and Colon-26 cells were treated with 1 μg/ml LL-37 or 100 ng/ml CRAMP and assessed for growth in soft agar by anchorage-independent colony formation assay. Scale bars = 500 μm. **(D and E)** Maximal colony size of HCT116, SW480 (D) and Colon-26 cells (E) in a soft-agar assay were determined. Results are mean α SEM, ***p < 0.001.

It has been well established that human cathelicidin LL-37 acts as a growth factor for human lung cancer and ovarian cancer cells [6, 7]. Based on the above data, it was hypothesized that cathelicidin may induce proliferation in colon cancer cell lines. The colon cancer cell lines, HCT116 and SW480, were incubated with different concentrations of LL-37; epidermal growth factor (EGF) was used as a positive control. The cell lines were treated for 96 h and a BrdU ELISA cell proliferation assay was subsequently performed. The proliferation of both colon cancer cell lines significantly increased after treatment with LL-37 at doses ranging from 50 ng/ml to 1 μg/ml (Fig. 2A). Cell proliferation was promoted at doses as low as 50 ng/ml, whereas cell proliferation decreased at higher doses (5 μg/ml to 50 μg/ml) (Fig. 2A).

An anchorage-independent colony formation assay was done to determine whether LL-37 promotes the ability of soft agar colony formation in colon cells. LL-37 significantly increased the ability of both colon cancer cell lines to grow in soft agar (Fig. 2C, 2D). The above experiments were repeated using the mouse colon cancer cell line, Colon-26. Colon-26 cells were treated with different doses of CRAMP (murine cathelicidin). Results were very similar to those obtained in human colon cancer cell lines treated with LL-37. CRAMP increased cell proliferation and colony formation in Colon-26 cells (Fig. 2B, 2C, 2E). Collectively, these results indicate that exogenous cathelicidin stimulates colon cancer cell proliferation and promotes anchorage-independent growth.

**Figure 3:**
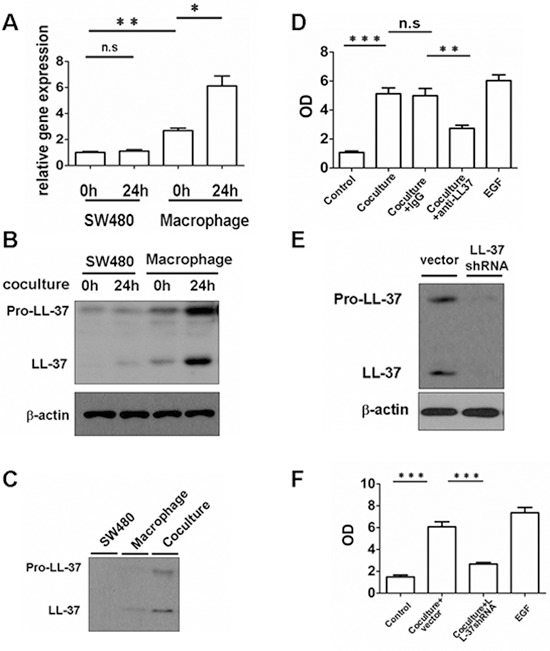
Macrophage-derived cathelicidin accelerates growth of colon cancer cells *in vitro* Human peripheral blood monocyte-derived macrophages and SW480 cells were coincubated. **(A and B)** Total RNA and protein from SW480 cells and macrophages were isolated 24 h after co-culture. The expression of hCAP18/LL-37 mRNA was measured by real-time PCR (A). The expression of hCAP18/LL-37 protein was analyzed by Western blot (B). **(C)** Western blots of cell supernatants from SW480, macrophages and co-culture at 24 h. **(D)** Cells were preincubated with 2 μg/ml anti-LL-37 or an IgG isotype control Ab (2 μg/ml) for 2 h. The cells were co-cultured in DMEM (10%FBS) for 96 h. One hundred ng/ml EGF was used as control. Cell proliferation was measured by ELISA (BrdU labeling) analysis. **(E)** Human macrophage-like cell line, U937, was transduced with LL-37 shRNA. After selection, expression of the indicated protein was analyzed. **(F)** SW480 cells were co-cultured with vector U937 cells or LL-37 shRNA U937 cells for 96 h and proliferation of SW480 cells was measured. Results are meanαSEM, *p < 0.05; **p < 0.01; ***p < 0.001; n.s, not significant.

Tumor-infiltrating myeloid cells in the tumor region expressed remarkably higher levels of hCAP18/LL-37, compared with tumor cells. Ex vivo tissue co-culture models were made, using human peripheral blood monocyte-derived macrophages and SW480 cells, in order to dissect the expression of cathelicidin. Coincubation of macrophages with SW480 cells induced hCAP18/LL-37 mRNA and protein (pro-LL-37 and cleaved LL-37) expression in macrophages, whereas hCAP-18/LL-37 expression in SW480 cells remained unchanged (Fig. 3A, 3B). In addition, hCAP18/LL-37 mRNA and protein expression was higher in macrophages than in SW480 cells (Fig. 3A, 3B). These results indicate that macrophages, and not SW480 cells, contribute to the release of the hCAP18/LL-37. Western blotting was performed on cell supernatants to assess the levels of pro-LL-37 and cleaved LL-37 after 24 h in co-culture. The levels of precursor and cleaved peptide were significantly higher in co-culture supernatants compared with either macrophages or SW480 cells (Fig. 3C). The proliferation capacity of SW480 cells had significantly increased after 96 h of coincubation with macrophages (Fig. 3D). As expected, addition of the neutralizing hCAP18/LL-37 antibody (anti-LL-37) to the co-culture significantly inhibited the proliferation of tumor cells (Fig. 3D). Silencing of hCAP18/LL-37 in U937 cells, a human macrophage-like cell line, via short hairpin RNA (shRNAs), significantly reduced the proliferation of SW480 cells (Fig. 3E, 3F). These findings suggest that LL-37 plays a direct role in promoting colon tumor cell proliferation and indicate that this peptide is required for promotion of cancer cell proliferation by macrophages.

### Neutralization of cathelicidin reduces colon tumor growth

**Figure 4:**
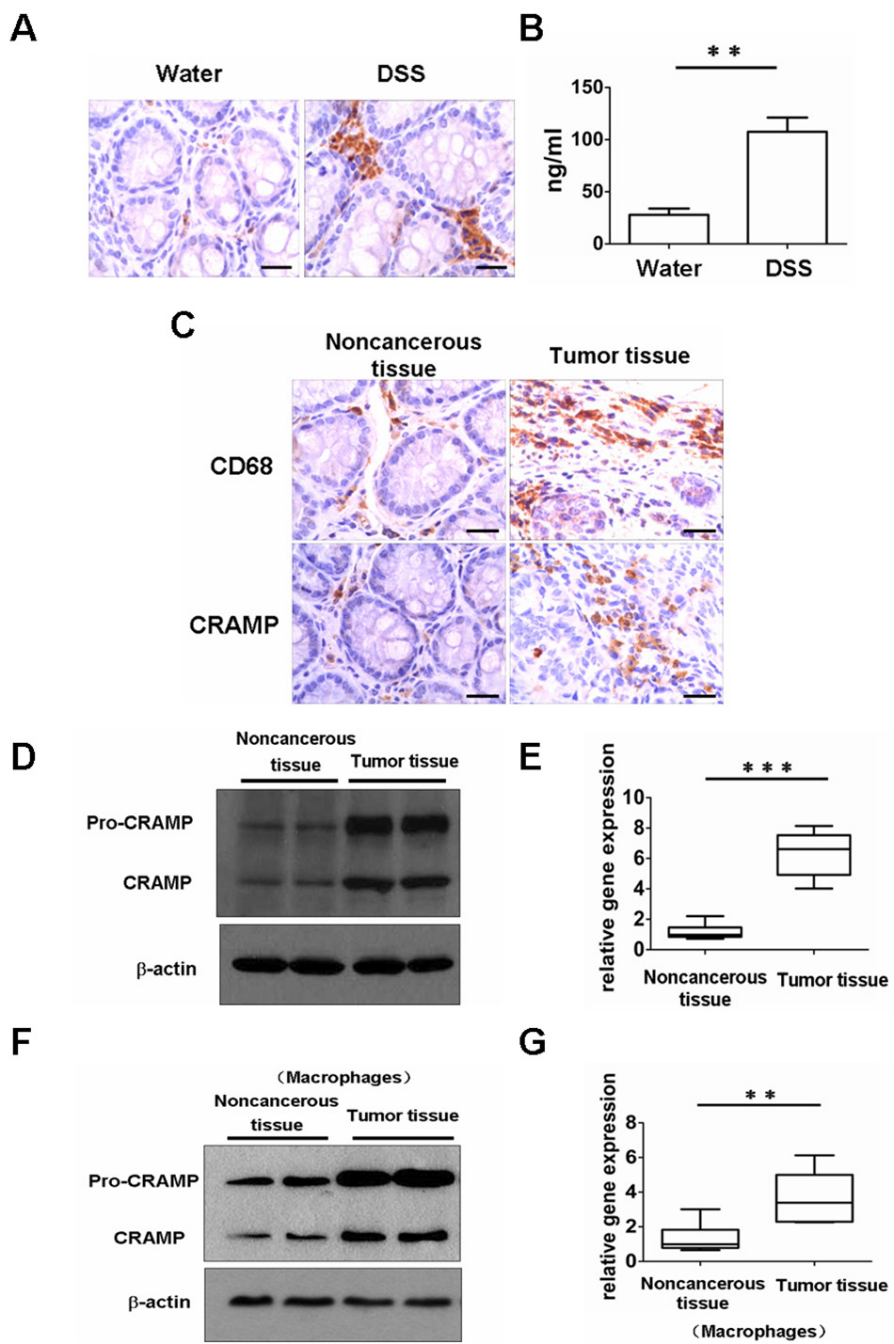
Expression of CRAMP and recruitment of macrophages in a mouse colitis-associated cancer model **(A)** The expression of CRAMP was examined in mice, 5 days after initiation treatment with 2.5% DSS (dextran sodium sulphate) or water, by immunohistochemistry of paraffin-embedded sections. Scale bars = 50 μm. **(B)** The serum levels of CRAMP after 2.5% DSS or water exposure were measured, on day 5, by ELISA. Results are meanαSEM (*n* = 5). **p < 0.01. **(C)** Immunohistochemical analysis of CRAMP or CD68 expression in AOM /DSS-treated noncancerous colon tissue and tumor-bearing colon tissue. Scale bars = 50 μm. **(D)** Expression of CRAMP proteins was examined by immunoblotting analysis in noncancerous colon tissue and colon cancer tissue. **(E)** The expression of CRAMP mRNA was measured by real-time PCR in noncancerous colon tissue and colon cancer tissue. Results are mean α SEM (*n* = 5), *n* = 5, ***p < 0.001. **(F)** The expression of CRAMP protein was analyzed in the macrophages isolated from noncancerous colon tissue and colon cancer tissue by western blot. **(G)** Induction of CRAMP mRNA was measured in macrophages isolated from noncancerous colon tissue and colon cancer tissue by real-time PCR. Results are mean α SEM (*n* = 5), *n* = 5, **p < 0.01.

**Figure 5:**
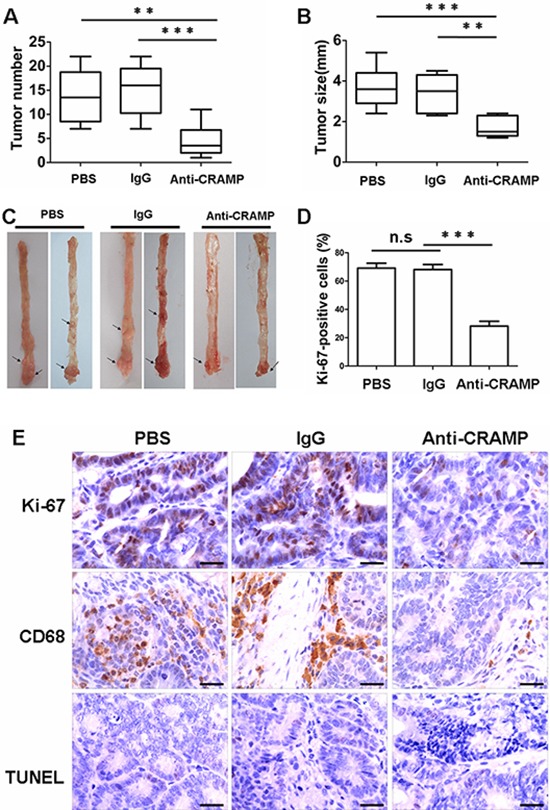
Neutralization of cathelicidin reduces colon tumor growth **(A and B)** Mice were subjected to an azoxymethane (AOM)-based colitis-associated cancer (CAC) induction protocol using three cycles of 2.5% dextran sodium sulphate (DSS) in drinking water. Beginning 80 days after treatment, mice received CRAMP-neutralizing antibody (anti-CRAMP), the IgG control antibody or PBS every 3 days for 30 days. Graphic representation of number (A) and size (B) of tumors obtained after surgical removal from PBS-, IgG- and anti-CRAMP-treated animals. Results are mean α SEM (*n* = 7). **p < 0.01, ***p < 0.001. **(C)** Colon tumors (arrows) in AOM/DSS-treated mice after PBS-, IgG- and anti-CRAMP treatment. **(D)** Percentage of Ki-67-positive tumor cells. Results are mean α SEM, (*n* = 7), ***p < 0.001; n.s, not significant. **(E)** Paraffin-embedded tumor-bearing colon sections were analyzed after staining with an anti-CD68 or Ki-67 antibody. Apoptotic cells were identified by TUNEL staining. Scale bars = 50 μm.

A colon cancer model was generated in order to investigate the effect of cathelicidin on colon cancer *in vivo*. Mice were injected with the procarcinogen azoxymethane (AOM) and then received three rounds of dextran sodium sulfate (DSS) exposure to elicit colitis. The expression of mouse cathelicidin (CRAMP) was higher in colonic tissues and the level of CRAMP was higher in the blood after exposure to DSS (Fig. 4A, 4B). All mice treated with AOM plus DSS developed tumors by day 80 (data not shown). Macrophage infiltration was observed in mouse tumor tissue, whereas macrophages were sparsely scattered in noncancerous mouse tissue, similar to results found in human cancerous and noncancerous tissues, respectively (Fig. 4C). CRAMP was highly expressed in the cytoplasm of infiltrating immune cells of the stroma (Fig. 4C). In contrast, there was a complete loss of CRAMP expression in colon tumor cells (Fig. 4C). These results were confirmed by immunoblot analysis and real-time PCR for CRAMP, in which the levels were found to be remarkably higher in tumor tissues (Fig. 4D, 4E). Importantly, CRAMP protein and CRAMP mRNA were also strongly expressed in macrophages that had been isolated from colon tumor tissue (Fig. 4F, 4G). Eighty days after treatment with AOM plus DSS, CRAMP neutralizing antibody (anti-CRAMP), IgG control antibody or PBS was administered to tumor-bearing animals for 30 days to investigate whether CRAMP contributes to colon cancer growth. Anti-CRAMP significantly reduced the number of macroscopic colon nodules and reduced maximal tumor size compared with the IgG control antibody and PBS (Fig. 5A, 5B). The tumor load in the colons of mice treated with anti-CRAMP was much lower compared to the colons of mice treated with IgG or PBS (Fig. 5C). The proliferation of tumor cells was then examined via immunohistochemical staining for Ki-67. Consistent with the changes in colon tumor burden, the number of cells expressing Ki-67 was significantly lower in tumors from mice treated with anti-CRAMP compared to mice treated with IgG or PBS (Fig. 5D, 5E). TUNEL staining was performed to determine whether the blocking of CRAMP had an impact on tumor cell apoptosis. There was no significant difference in colon tumor cell apoptosis between the group treated with anti-CRAMP and the group treated with IgG or PBS (Fig. 5E). Thus, cathelicidin appears to play a critical role in the promotion of colon tumor growth, most likely through stimulating the proliferation of malignant cells. In addition, the macrophage infiltration into colon tumors was much lower in mice treated with anti-CRAMP than in mice treated with IgG or PBS (Fig. 5E), indicating that cathelicidin plays a critical role in directing immune cells into tumor tissues.

### Wnt/β-catenin signaling mediates tumor growth promoted by cathelicidin

**Figure 6:**
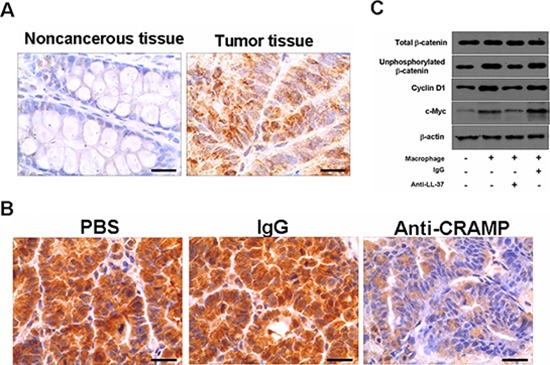
Cathelicidin-blocking affects the activation of the Wnt/β-catenin signaling pathway in colon cancer cells **(A)** Immunohistochemical analysis of unphosphorylated β-catenin in noncancerous and colon cancer tumor tissue from humans. Scale bars = 50 μm. **(B)** Expression of unphosphorylated β-catenin in colon tumor sections of PBS-, IgG- and anti-CRAMP-treated mice. Scale bars = 50 μm. **(C)** Anti-LL-37 antibody or IgG was applied to SW480 cells in coculture with human peripheral blood monocyte-derived macrophages. After 72 h, the indicated proteins in SW480 cells were analyzed by immunoblotting.

As Wnt/β-catenin signaling mediates tumor proliferation [3], we examined β-catenin phosphorylation in the colon cancer cells. As shown in Fig. 6A, the protein level of unphosphorylated β-catenin in the human colon cancer cells was significantly higher than that in noncancerous control tissues (60/68 cases had positive tumors). The accumulation of unphosphorylated β-catenin was corresponding with the expression of hCAP-18/LL-37 in tumor tissues (Fig. 1A). The tumor tissues derived from mouse models that received anti-CRAMP neutralizing antibody had markedly lower levels of unphosphorylated β-catenin in tumor cells than the control tissues derived from PBS or isotype IgG-treated cancer mice (Fig. 6B). Similarly, the addition of HCAP18/LL-37 neutralizing antibody significantly suppressed the accumulation of unphosphorylated β-catenin in SW480 cells, which were co-cultured with macrophages (Fig. 6C). Moreover, the expression of β-catenin targeting gene products, c-Myc and cyclin D1, in SW480 cells was also reduced by anti-LL-37 (Fig. 6C).

**Figure 7:**
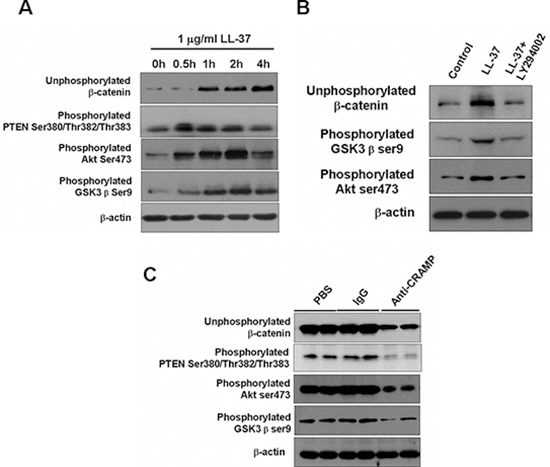
LL-37 activates β-catenin through PTEN/Akt/GSK3β pathways **(A)** SW480 cells were treated with 1 μg/ml LL-37 for the indicated times. Phosphorylation of different proteins was detected by Western blot analysis. **(B)** SW480 cells were exposed to 1 μg/ml LL-37, with or without 10 μM LY294002, for 2 h. Expression of the indicated proteins was detected by immunoblotting analysis. **(C)** Expression of indicated proteins was examined by Western blot analysis in isolated tumors of AOM /DSS-mice treated with PBS, IgG- or anti-CRAMP.

GSK3β and Akt proteins are known to regulate Wnt/β-catenin signaling [3, 16]. GSK3β phosphorylation on Ser9 and Akt phosphorylation on Ser473 were significantly increased in SW480 cells that had been treated with LL-37 for 4 h (Fig. 7A). Interestingly, the unphosphorylated β-catenin was accumulated in association with the phosphorylation of GSK3β and Akt (Fig. 7A). The application of the PI3K/Akt inhibitor, LY294002, inhibited the LL-37-induced accumulation of unphosphorylated β-catenin, as well as the phosphorylation of GSK3β in SW480 cells (Fig. 7B).

Phosphatase and tensin homolog (PTEN) is a tumor suppressor negatively regulating PI3K/Akt signaling and phosphorylation in the C terminus decreases stability leading to more rapid degradedation by the proteasome [17]. To test whether PTEN is involved in the regulation of Akt activation by cathelicidin, we observed that phosphorylation of PTEN at Ser380, Thr382 and Thr383 in cultured colon cancer cells could be induced by LL-37 exposure (Fig. 7A) or in cancer cells in the cancer animal models (Fig. 7C). In the mouse model, anti-CRAMP antibody treatment could block phosphorylation of all PTEN, Akt and GSK3β (Fig. 7C). These results indicate that inhibition of PTEN by phosphorylation could activate PI3K/Akt signaling and subsequent GSK3β inhibition, which in the end activate Wnt/β-catenin signaling and promote the growth of colon tumor cells.

## DISCUSSION

The infiltrating immune cells have formed the key part of tumor microenvironment in the colon cancer. How these immune cells affects tumor genesis still remains unclear [1, 18, 19]. Our study demonstrates that cathelicidin is strongly expressed and secreted by the infiltrating macrophages and significantly contributes to the growth of colon cancer through activating Wnt/β-catenin signaling in cancer cells.

Our study observed dramatical expression of cathelicidin in the cancerous colon tissue in both CRC patients and CRC mouse models. The immune cells, especially macrophages infiltrating the tumor, instead of the tumor and colonic epithelial cells are the major source of cathelicidin. This observation was confirmed by the co-culture of macrophages and tumor cells, which showed increased cathelicidin expression in macrophages, but not in tumor cells. Our observation is also in line with an earlier report on the lung cancer, in which sufficient macrophages are recruited to tumor areas and secret cathelicidin [6]. However, our results are different from Cho group's report that the expression of cathelicidin in colon cancer was lower than in noncancerous colon tissue and colonic epithelial cells were the major cells expressing cathelicidin [20].

Cathelicidin was originally showed with its antimicrobial activity. Cathelicidin shields from skin and urogenital endothelium during infections and protects against tuberculosis [8, 21, 22]. Recently, cathelicidin LL-37 has been shown to be over-expressed in lung, breast, ovarian and prostate cancers and showing tumor-promoting effects [12, 23, 24]. In our study, we demonstrated that the tumor-promoting effect of cathelicidin was dose-dependent: i) cathelicidin at the physiological level or even lower doses (1 μg/ml for human colon cancer cells; 100 ng/ml for mouse colon cancer cells) promoted tumor growth in cell culture; ii) the inhibition of cathelicidin signaling in animal models by neutralizing the endogenous cathelicidin with antibodies reduced tumor growth; and iii) cathelicidin at higher concentrations (LL-37: 50 μg/ml; or CRAMP: 1000 ng/ml) killed the tumor cells, which could explained the previous observations from Isogai [25] and Cho [20, 26] that cathelicidin at high doses of (Isogai: 10 to 40 μg/ml or Cho: 20 to 60 μmol/l) decreased the proliferation of colon cancer cells by inducing apoptosis and autophagy. Moreover, cathelicidin could serve as a chemokine of inflammatory cells including monocytes, neutrophils and macrophages [6, 27]. Thus, the expression of cathelicidin in immune cells builds a positive feedback loop in formation of the inflammatory tumor microenvironment.

The development of colon cancer has been related to numerous genetic mutations and various altered signaling pathways, including the Wnt signaling pathway [28]. In colon cancer, increased β-catenin accumulation has been observed in the invasive front of tumor [16]. In this study, we demonstrated that LL-37 enhenced the Wnt/β-catenin signaling in CRC tumor cells. β-catenin is the central player in the Wnt/β-catenin pathway and the levels of β-catenin are tightly regulated [29]. In the absence of Wnt, cytoplasmic β-catenin is phosphorylated on several serine and threonine residues by a destruction complex containing glycogen synthase kinase-3β (GSK3β), casein kinase1 (CK1), axin and adenomatous polyposis coli (APC), which leads to ubiquitin-proteasome-mediated degradation of β-catenin [30, 31]. Upon binding of Wnt engage their receptors on the cell surface, the destruction complex including GSK3β is inhibited. As a result, β-catenin is stabilized and enters the nucleus, where the expressions of a broad range of target genes, such as CyclinD1 and c-Myc, are modulated [31, 32]. A study by Castellone et al. suggested a mechanism in which inflammatory PGE2 increased unphosphorylated β-catenin levels by phosphorylation of an inhibitory residue in GSK3β, via Akt, in colon cells [33]. In the current study, LL-37 treatment activated Akt in colon cancer cells through a PI3K-dependent pathway. In turn, LL-37 inhibited GSK3β kinase activity by phosphorylating serine 9, and subsequently activated β-catenin. The possibility that cathelicidin alters PTEN phosphorylation was examined in order to further elucidate the mechanism by which cathelicidin activates Akt. PTEN is a major negative regulator of the PI3K/Akt signaling pathway, which dominates the activation of Akt by dephosphorylating PIP3 to generate PIP2 [34]. PTEN possesses a carboxy-terminal noncatalytic regulatory domain containing three phosphorylation sites (Ser380, Thr382 and Thr383), which regulate its stability and have been implicated in controlling PTEN activity [35]. In the present study, there was a significant increase in PTEN phosphorylation in SW480 colon cancer cells following stimulation by human peptide, LL-37. Moreover, anti-CRAMP antibody was able to inhibit the phosphorylation of PTEN, as well as Akt and GSK3β in the mouse model. These results outline a novel mechanism for the effects of cathelicidin in colon cancer cells and the promotion of colon cancer growth through a PTEN/PI3K/Akt/GSK3β/β-catenin signaling axis.

In conclusion, our study demonstrates that cathelicidin is upregulated in immune cells infiltrating the colon cancer tissue. Cathelicidin promotes tumor growth through activating PTEN/PI3K/Akt/Wnt/β-catenin signaling pathway. Thus, our findings contribute to a better understanding of the pathogenic mechanism of tumor-infiltrating immune cells, and could offer a novel molecular target for the colon cancer therapy.

## MATERIALS AND METHODS

### Patients and specimens

Freshly resected tumor specimens and corresponding noncancerous colon mucosal tissues were immediately formalin-fixed and paraffin-embedded. Samples from 68 patients who did not receive radiation or chemotherapy before the surgical resection of the primary colon lesion were included for immunohistochemical analysis. Blood samples were drawn from the 68 patients before surgery and 1 month after surgery. Control blood samples were obtained from 30 healthy donors. The study was performed according to the rules and regulations of and was approved by the local research ethical committees (Internal Review and the Ethics Boards of the Tongji Hospital, Tongji University). All subjects gave written informed consent.

### Animal tumor induction and analysis

Female C57BL/6 mice were obtained from the National Rodent Laboratory Animal Resource (Shanghai Branch, PRC) and maintained in a pathogen-free Central Animal Facility of the Tongji Hospital of Tongji University. This study was carried out in strict accordance with the recommendations in the Guidelines for the Care and Use of Laboratory Animals of the National institutes of Health. All animal experiments were approved by the Tongji Hospital of Tongji University Ethics Committee on the Use and Care of Animals. All surgery was performed under sodium pentobarbital anesthesia and all efforts were made to minimize suffering.

The colitis-associated colon cancer model was induced as described previously [36]. Briefly, on day 1, mice were injected intraperitoneally (i.p.) with 12.5 mg/kg azoxymethane (AOM) (Sigma-Aldrich, St. Louis, MO, USA) and maintained on regular diet and water for 5 days. After 5 days, mice received water with 2.5% dextran sulfate sodium (DSS; molecular weight 35,000–50,000 kDa; MP Biomedicals, Santa Ana, CA, USA) for 5 days. After 5 days of treatment with 2.5% dextran sulfate sodium, mice were maintained on regular water for 14 days and then subjected to 2 more DSS treatment cycles. On day 80, mice were injected with 10 mg/kg i.p of nonspecific rabbit IgG antibody (Cell Signalling Technology, Danvers, MA, USA), rabbit anti-CRAMP (Pineda-Antikörper-Service, Berlin, Germany) or PBS. Antibodies or PBS were given every three days for the duration of the experiment. On day 110, mice were euthanized and the colons were removed and examined histologically or by western blot. Macroscopic tumors were counted and measured with a caliper. Mice were euthanized after 5 days of 2.5% DSS or water treatment to study the expression of CRAMP. The serum was used for enzyme-linked immunosorbent assay analysis (ELISA).

### Cell culture

The human colon cancer cell lines, HCT116 and SW480, the mouse colon cancer cell line, Colon-26 and the human macrophage-like cell line, U937, were obtained from the American Type Culture Collection. Cells were cultured in Dulbeccos's modified Eagles medium (DMEM) (Hyclone laboratories. Inc, South, UT, USA) supplemented with 10% fetal calf serum (FCS) (Invitrogen, Grand Island, NY, USA), 100 U/ml penicillin and 100 U/ml streptomycin (Hyclone laboratories. Inc.). Cell cultures were performed at 37°C in humidified air with 5% CO_2_. For monocytic differentiation, U937 cells were cultured in the presence of 10 nM phorbol-12myristat-13acetat (Sigma-Aldrich) for 48 h. The PI3K/Akt inhibitor, LY294002 (10 μM, Cell Signaling Technology) was added as indicated, 1 h before LL-37 (Invivogen, San Diego, CA, USA) stimulation.

### Generation of human peripheral blood monocytederived macrophages

Institutional approval from the local research ethical committees (Internal Review and the Ethics Boards of the Tongji Hospital, Tongji University) was obtained prior to conducting the study. Human peripheral blood monocyte-derived macrophages were generated as previously described [6]. Briefly, human peripheral blood mononuclear cells (PBMC) from healthy blood donors from the blood bank of the Tongji Hospital of Tongji University were isolated from buffy coats by Ficoll-Paque PLUS (GE Healthcare, Uppsala, Sweden) density centrifugation. PBMCs were allowed to adhere to culture flasks for 1 h at 37°C in DMEM supplemented with 1% human serum, after which the nonadherent cells were removed by vigorous washing with PBS. Adherent cells were cultured in 20 ml DMEM (10% FCS) supplemented with 50 ng/ml macrophage colony stimulating factor (M-CSF) (eBioscience, San Diego, CA, USA) for 7 days to allow differentiation to macrophages.

### Isolation of macrophages from mouse tumors or noncancerous colon tissues

Mouse primary macrophages were generated as described previously [36]. Briefly, tissue was chopped into small pieces and placed in 1 ml enzyme-mixture buffer (400 U/ml collagenase type IV, 0.05 mg/ml collagenase type I, 0.025 mg/ml hyaluronidase, 0.01 mg/ml DNase I, 0.2 U/ml soybean trypsin inhibitor (all five from Sigma-Aldrich) dissolved in DMEM + 10% FBS) and incubated for 2 h at 37°C. The mixture was then pipetted up and down with a 1 ml pipette to get an easily flowing single cell suspension. The cell suspension was then centrifuged and the cell pellet was resuspended in MACs buffer. Anti-CD11-microbeads (5 μl beads/10^7^ cells) were then added and the suspension was incubated at 8°C for 15 min. CD11b^+^ cells were purified on LS columns, using the MidiMACS system, according to the manufacturer's instructions (Miltenyi Biotec GmbH, Bergisch Gladbach, Germany). Cells were cultured in DMEM supplemented with 10% FBS for 1 h at 37°C. Non-adherent cells were removed by vigorous washing with phosphate-buffered saline and the purity of the prep was confirmed by morphology and staining with macrophage specific marker, CD68.

### Co-cultivation of colon cancer cells and macrophages

For coculture studies with cancer cells and macrophages, cancer cells were seeded into the bottom of multi-well cell culture plates and macrophages were placed in transwell inserts (0.4 μm, Corning Incorporated, Corning, NY, USA) with a membrane permeable for liquids, but not for cells. Cells were incubated overnight in DMEM supplemented with 10% FBS. The transwells were inserted into the wells of a multi-well culture plate and cultured for the indicated time. Neutralizing antibody (Clone # mAb 3D11, Hycult Biotech, The Netherlands) was added at 2 μg/ml for hCAP18/LL-37 neutralization.

### BrdU ELISA cell proliferation assay

Colon cancer cell proliferation was determined using a commercially available Cell Proliferation ELISA, BrdU (colorimetric) Kit (Roche, Mannheim, Germany), as previously described [7]. For cathelicidin stimulation, the cells were stimulated with 0-50 μg/ml LL-37 (Invivogen) or 0-1000 ng/ml CRAMP (AnaSpec, Fremont, CA, USA) for 96 h; human EGF (hEGF) (Invitrogen) or mouse EFG (mEGF) (Invitrogen) was used as control. For coculture experiments, after 4 days coculture with macrophages, transwell inserts (containing macrophages) were removed, tumor cell supernatants were aspirated and growth media, containing 10 μM BrdU, was added. Tumor cells were incubated for an additional 2 h at 37°C, after which cell proliferation was measured.

### Anchorage-independent colony formation assay

24-well plates were precoated with 0.8ml of 0.5% (w/v) agar containing 10% (v/v) FBS as a bottom layer. Cell lines were suspended in 0.6 ml 0.3% (w/v) agar containing 10% (v/v) FBS and plated onto the bottom layer. Cathelicidins were used at the indicated concentrations in the media. Colony forming efficiency was determined 2 to 3 weeks after plating and cultivation in a humidified 5% CO2 atmosphere at 37°C. The colonies were visualized after staining with 0.005% crystal violet.

### Small interfering RNA interference

Log growth U937 cells were washed 1 time with PBS and resuspended at 2×10^7^ cells/ml in 1 ml of Gene Pulser electroporation buffer reagent (Bio-Rad, Hercules, CA, USA), mixed with 20 mg of LL-37 RNAi plasmids or mock RNAi plasmids (both from Santa Cruz Biotechnology, Santa Cruz, CA, USA). Electroporations were performed using a Gene-Pulser (Bio-Rad) at 280 V and 960 mF in 0.4 cm cuvette (Bio-Rad). The samples were transferred to culture flasks containing complete DMEM medium with 10% FCS in 25 cm^2^ and incubated at 37°C in 5% CO_2_. After 48 h, the growth medium was changed and puromycin (Invitrogen) was added at a concentration of 5 mg/ml. The culture medium was switched with fresh growth medium (containing 5 mg/ml puromycin), every 4 days. After 4 weeks, positive polyclonal populations (pools) were identified based on western blot analysis for LL-37 expression. Individual positive clones were eventually isolated via limiting dilution analysis in 96-well plates.

### ELISA assay for cathelicidin

Samples of serum from patients or mice were analyzed by hCAP18/LL-37 or CRAMP ELISA (USCN life science, INC. Houston, TX, USA), respectively, according to the manufacturer's instructions.

### RNA isolation and real-time PCR

Total RNAs were isolated from microdissected tumors and noncancerous colon tissues or cells, according to the manufacturer's recommendations, using an RNeasy plus mini kit (Qiagen, Santa Clarita, CA, USA). Real time PCR reaction mixtures have been described previously [3]. Briefly, cDNA was synthesized by reverse transcription reaction using the First Strand cDNA synthesis kit (Invitrogen). Real-time PCR was performed using the QPCR SYBR Green Mix (Bio-Rad) on an AB 7300 Real time PCR system machine (AB Applied Biosystems, Singapore). The following PCR primers were used: human β-actin, 5′-AGCCTCGCCTTTGCCGA-3′ and 5′-CTGGTGCCTGGGGCG-3′; mouse β-actin, 5′-AGCCTCGCCTTTGCCGA-3′ and 5′-CTGGTGCCTGGGGCG-3′; hCAP18/LL-37, 5′-TGGGCCTGGTGATGC CT-3′ and 5′-CGATGTTCCTTCGACAGGAAGC-3′; mouse CRAMP, 5′-AATTTT CTTGAACCGAAAGGGC-3′ and 5′-TGTTTTCTCAGATCCTTGGGAGC-3′. Specificity of RT-PCR was controlled by ‘no reverse transcription’ controls and melting curve analysis. Quantitative PCR results were obtained using the DDCT (cycle threshold) method. Data were normalized to β-actin levels in each sample.

### Immunohistochemical analyses

Immunohistochemistry was performed as described before [6]. The following primary antibodies were used for immunohistochemical analysis: mouse anti-Ki-67 (Abcam, Cambridge, UK), rabbit anti-CD68 (for mouse, Abbiotec, San Diego, CA, USA), mouse anti-CD68 (Dako, Carpinteria, CA, USA), rabbit anti-CRAMP (Pineda-Antikörper-Service, Berlin, Germany), rabbit anti-hCAP18/LL-37 (Santa Cruz Biotechnology) and mouse anti-unphosphorylated β-catenin (Millipore, Temecula, CA, USA). Secondary antibody incubation and staining were performed using the EnVision®+ System–HRP (DAB) kit (Dako), according to manufacturer's recommendations. TUNEL staining was performed using the DeadEnd Colorimetric TUNEL System kit (Promega, Madison, WI, USA). The number of Ki-67-positive tumor cells and the total number of tumor cells were enumerated in six microscopic fields of a randomly selected tumor and the mean value was calculated as the percentage of Ki-67-positive tumor cells.

### Preparation of cell total protein extract and western blotting

In all, 10mg of microdissected tumor tissue was homogenized in 500 ml cell lysis buffer (Cell Signalling Technology) using a rotor-stator homogenizer. Western blot analysis was performed as described earlier [7]. Briefly, 30 mg total protein extracts were loaded on 10% SDS polyacrylamide gels, subjected to electrophoresis, and blotted onto Hybond-C Extra membranes (Amersham Bioscience, Buckinghamshire, UK). The cell supernatants were first concentrated to 1/10 of their original volume by vacuum centrifugation and then subjected to 4–12% NU/PAGE gradient gels (Invitrogen). Primary antibodies used for western blot analysis included: mouse anti-cyclin D1, rabbit anti-c-myc, rabbit anti-total β-catenin, rabbit anti-GSK3β (pSer9), rabbit anti-Akt (pSer473), rabbit anti-PTEN (p Ser380/pThr382/pThr383 (all six from Cell Signaling Technology), mouse anti-unphosphorylated β-catenin (Millipore) and mouse anti-β-actin (Sigma Aldrich). HRP-conjugated goat anti rabbit (Santa Cruz) or rabbit anti-mouse (Dako) was used as the secondary antibody.

### Statistical analysis

Data are expressed as mean ± SEM. Comparisons between groups were analyzed by t-test (two-sided). A *P*-value of < 0.05 was considered statistically significant.
